# Supplementary material for: Overexpression of GmbZIP59 Confers Broad-Spectrum Stress Resistance in Arabidopsis thaliana and Rice (Oryza sativa)
Source: Plants (Basel). 2025 Oct 30;14(21):3326. doi: 10.3390/plants14213326 (PMC12608880; doi:10.3390/plants14213326)
Supplement: Supplementary file 1 [file plants-14-03326-s001.zip › Supplementary Figures.pdf]

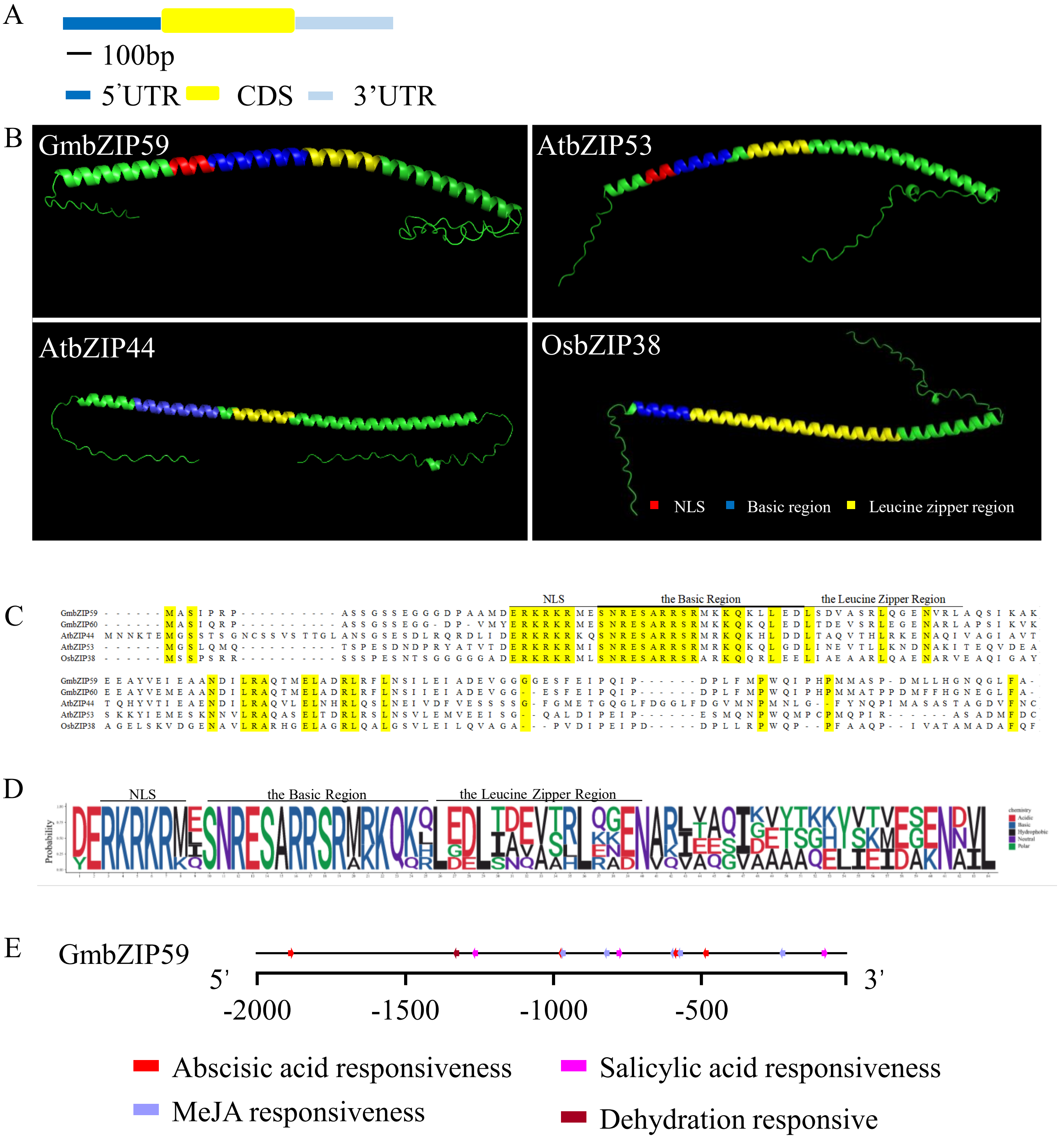

Supplemental Figure S1. Bioinformatics analysis of GmbZIP59. A Gene structure analysis. B Protein structure analysis. C Multiple sequence alignments of the conserved basic region and leucine zipper region among GmbZIP59, GmbZIP60, AtbZIP44, AtbZIP53, and OsbZIP38. D Protein sequence logo analysis. E Predicted *cis*-elements in *GmbZIP59* promoter regions.

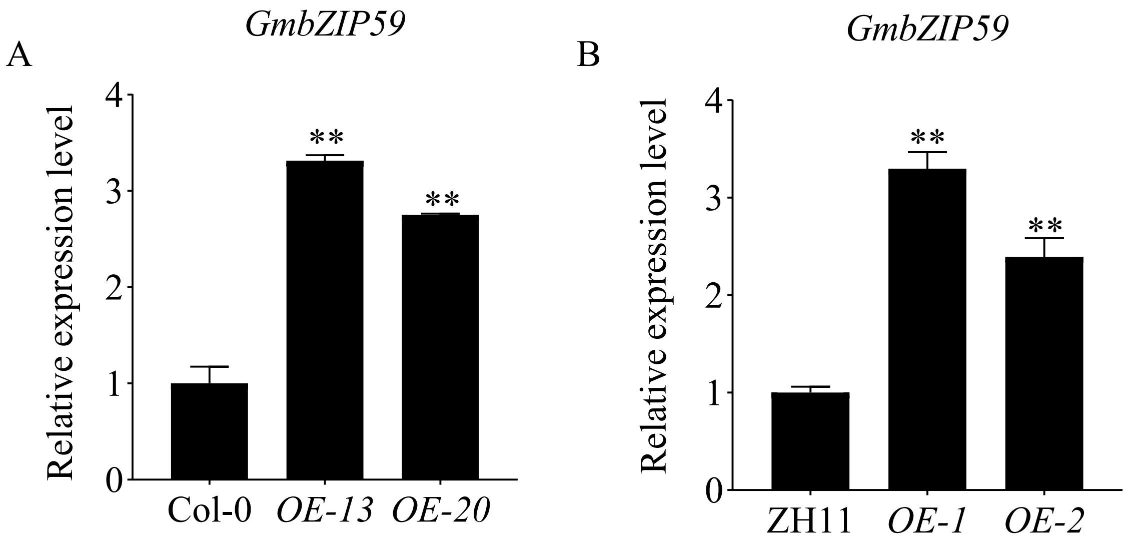

Supplemental Figure S2. Analysis of *GmbZIP59* expression levels in transgenic *Arabidopsis* and rice plants. (A) Relative expression level of *GmbZIP59* in Col-0, *OE-13*, and *OE-20 Arabidopsis* transgenic lines. (B) Relative expression level of *GmbZIP59* in William82, *OE-1*, and *OE-2* rice transgenic lines. Error bars represent ± SD from three biological replicates. Asterisks indicate statistically significant differences based on a Student's *t-test* (** $p < 0.01$; * $p < 0.05$).

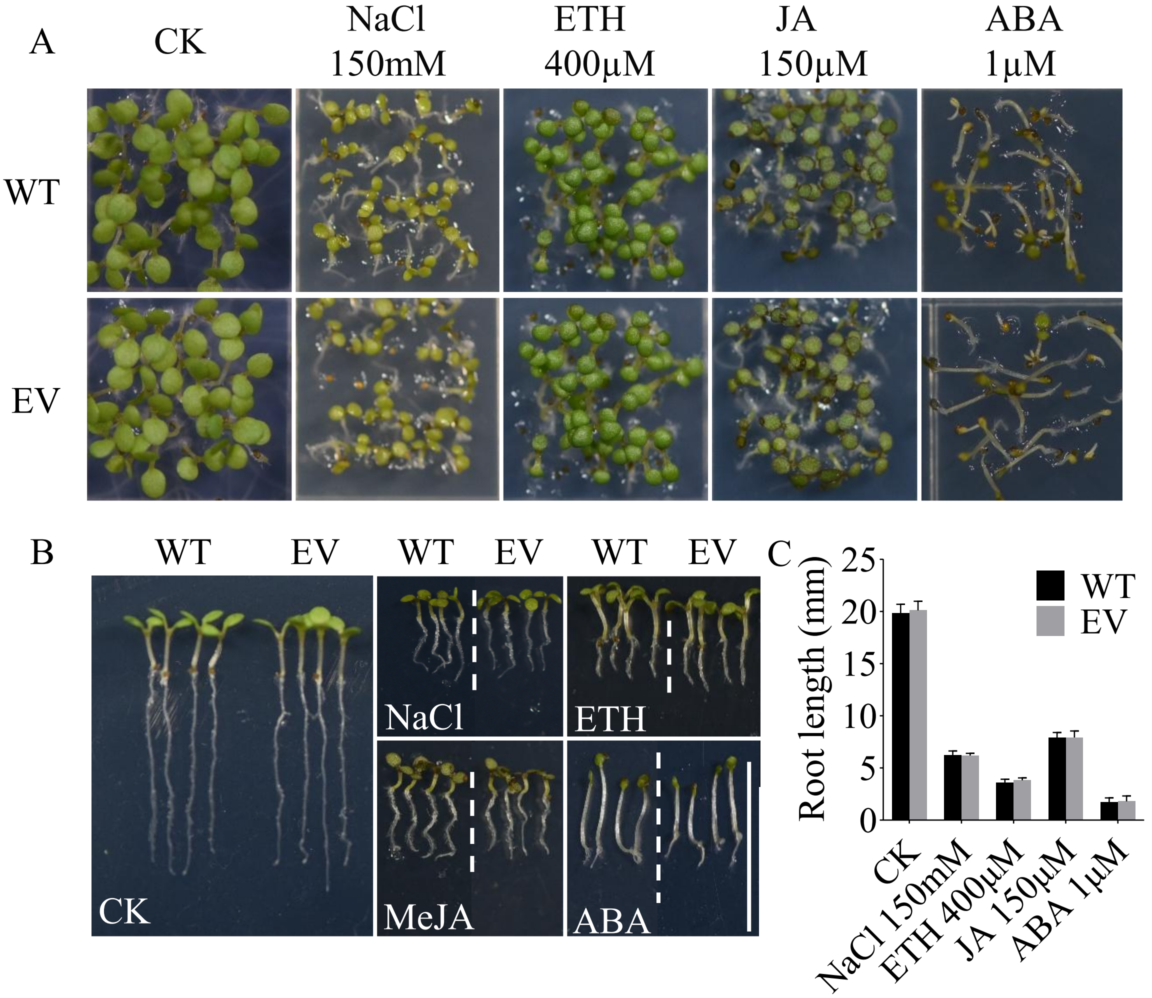

Supplemental Figure S3. Phenotypic analysis of empty vector (EV) in *Arabidopsis* plants in response to salt and plant hormones treatment. (A) All the seeds were germinated on the 1/2 Murashige and Skoog Medium (MS) medium under normal conditions or supple-mented with CK (Control check), NaCl (150 mM), ETH (400 uM), MeJA (200 uM), and ABA (1.0 uM) for 1 week (Scale bar, 1 cm). (B) Calculation of the seedlings' root length. Abscisic acid (ABA), Methyl jasmonic acid (MeJA) and Ethylene (ETH), Asterisks indicate significant differences for the indicated comparisons based on a Student's *t-test* (** $p < 0.01$; $0.01 <$ * $p < 0.05$).

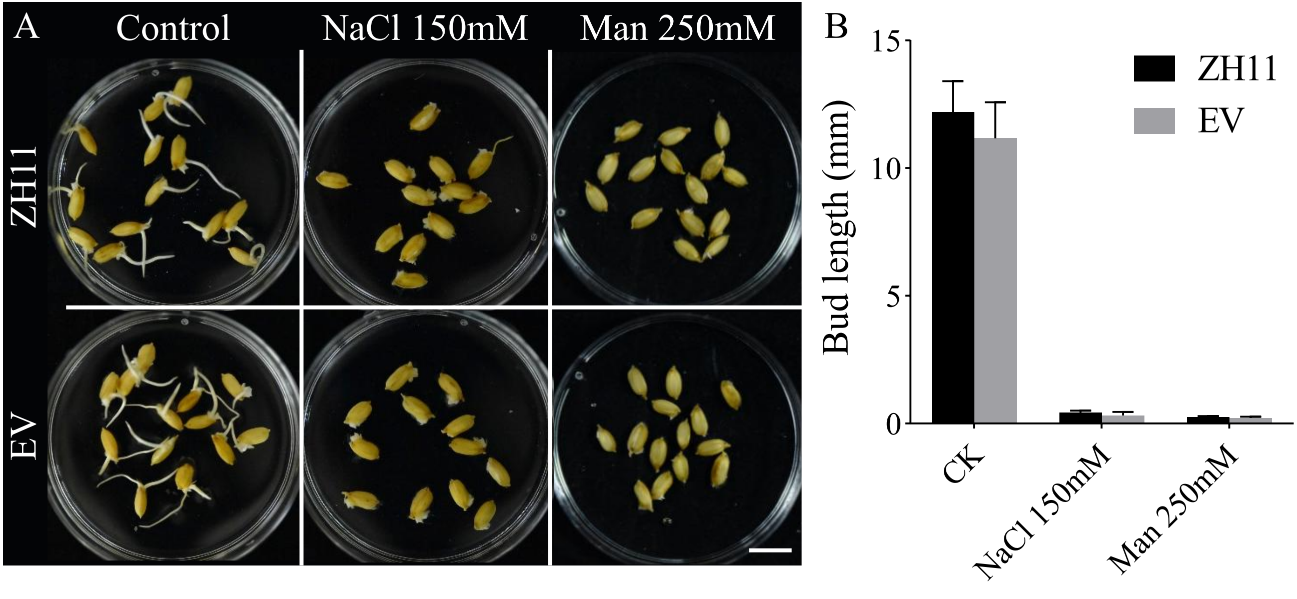

Supplemental Figure S4. Phenotypic analysis of empty vector (EV) in *Arabidopsis* plants in response to salt and plant hormones treatment. (A) Seeds were germinated under control or subjected to NaCl (150 mM) and mannitol (250 mM) treatments for 4 days. Scale bar, 1 cm. (B) Measurement of the seedling bud lengthErrors bars represent ± SD of three biological repli-cates. Asterisks denote significant differences between the indicated comparisons based on a student's *t-test* (** $p < 0.01$; $0.01 < * p < 0.05$).
